# Sphingosine 1-Phosphate-Induced ICAM-1 Expression via NADPH Oxidase/ROS-Dependent NF-κB Cascade on Human Pulmonary Alveolar Epithelial Cells

**DOI:** 10.3389/fphar.2016.00080

**Published:** 2016-03-31

**Authors:** Chih-Chung Lin, Chien-Chung Yang, Rou-Ling Cho, Chen-Yu Wang, Li-Der Hsiao, Chuen-Mao Yang

**Affiliations:** ^1^Department of Anesthetics, Chang Gung Memorial Hospital at LinkouTaoyuan, Taiwan; ^2^College of Medicine, Chang Gung UniversityTaoyuan, Taiwan; ^3^Department of Physiology and Pharmacology and Health Aging Research Center, College of Medicine, Chang Gung UniversityTaoyuan, Taiwan; ^4^Department of Traditional Chinese Medicine, Chang Gung Memorial Hospital at LinkouTaoyuan, Taiwan; ^5^Research Center for Industry of Human Ecology and Graduate Institute of Health Industry Technology, Chang Gung University of Science and TechnologyTaoyuan, Taiwan

**Keywords:** sphingosine-1-phosphate, intercellular adhesion molecule-1, lung inflammation, transcription factor, monocyte adhesion

## Abstract

The intercellular adhesion molecule-1 (ICAM-1) expression is frequently correlated with the lung inflammation. In lung injury, sphingosine-1-phosphate (S1P, bioactive sphingolipid metabolite), participate gene regulation of adhesion molecule in inflammation progression and aggravate tissue damage. To investigate the transduction mechanisms of the S1P in pulmonary epithelium, we demonstrated that exposure of HPAEpiCs (human pulmonary alveolar epithelial cells) to S1P significantly induces ICAM-1 expression leading to increase monocyte adhesion on the surface of HPAEpiCs. These phenomena were effectively attenuated by pretreatments with series of inhibitors such as Rottlerin (PKCδ), PF431396 (PYK2), diphenyleneiodonium chloride (DPI), apocynin (NADPH oxidase), Edaravone (ROS), and Bay11-7082 (NF-κB). Consistently, knockdown with siRNA transfection of PKCδ, PYK2, p47*^phox^*, and p65 exhibited the same results. Pretreatment with both Gq-coupled receptor antagonist (GPA2A) and Gi/o-coupled receptor antagonist (GPA2) also blocked the upregulation of ICAM-1 protein and mRNA induced by S1P. We observed that S1P induced PYK2 activation via a Gq-coupled receptor/PKCδ-dependent pathway. In addition, S1P induced NADPH oxidase activation and intracellular ROS generation, which were also reduced by Rottlerin or PF431396. We demonstrated that S1P induced NF-κB p65 phosphorylation and nuclear translocation in HPAEpiCs. Activated NF-κB was blocked by Rottlerin, PF431396, APO, DPI, or Edaravone. Besides, the results of monocyte adhesion assay indicated that S1P-induced ICAM-1 expression on HPAEpiCs can enhance the monocyte attachments. In the S1P-treated mice, we found that the levels of ICAM-1 protein and mRNA in the lung fractions, the pulmonary hematoma and leukocyte count in bronchoalveolar lavage fluid were enhanced through a PKCδ/PYK2/NADPH oxidase/ROS/NF-κB signaling pathway. We concluded that S1P-accelerated lung damage is due to the ICAM-1 induction associated with leukocyte recruitment.

## Introduction

Sustained stimulation and inflammation in respiratory system are pivotal events in pathogenic progressing of asthma or other chronic obstructive pulmonary diseases. To regulate inflammatory responses, not only initiated complicated interaction of the intracellular molecules but also recruited serious intercellular interactions such as lung resident cells, vascular endothelium and circulating polymorphonuclear cells (PMNs) (Lin et al., 2015). Previous studies indicate that pro-inflammatory cytokine-induced expression of adhesion molecules on the endothelial cells surface is implicated in the inflammatory responses and facilitate (or guidance) PMNs infiltration to the inflammatory area. (Kim et al., 2008; Lee and Yang, 2012). In the classification of adhesion molecules, these molecules were divided into two groups: the Ig superfamily and selectins. ICAM-1 and VCAM-1 are belong to the Ig superfamily (Lee and Yang, 2013). The inducible endothelial adhesive glycoprotein, ICAM-1, is identified on several types of cells (Albelda et al., 1994; Rahman and Fazal, 2009). In the regulation of transendothelial migration, ICAM-1 expression can mediate the tight adhesiveness of PMNs. PMNs were arrested at non-junctional locations to penetrate the vascular endothelium barrier and then attached on the resident cells (Yang et al., 2005; Lee et al., 2013b).

Sphingosine 1-phosphate (S1P, a bioactive sphingolipid metabolite) plays important roles in allergic responses, including asthma, and anaphylaxis (Ammit et al., 2001). S1P regulates numerous cellular responses, including motility, and cytoskeletal rearrangements, formation of adherent junctions, proliferation, survival, angiogenesis, and the trafficking of several types of cells (Ryan and Spiegel, 2008; Li et al., 2009). These myriad effects are partly elicited by binding of S1P to a family of five G protein–coupled receptors (S1PRs), termed S1PR1–5. Moreover, S1P has been shown to induce lung injury and inflammation (Sun et al., 2013). In addition, S1P has been also shown to induce ICAM-1 or VCAM-1 expression in various cell types (Milara et al., 2009; Costello et al., 2011). However, the mechanisms of S1P-regulated ICAM-1 expression in human pulmonary alveolar epithelial cells (HPAEpiCs) are not completely understood. Thus, to clarify the mechanisms of ICAM-1 induction by S1P on pulmonary alveolar epithelium was proposed to be a new therapeutic target in the respiratory diseases.

PKCδ was first identified from mammalian cDNA libraries and classified to be a member of novel PKC subfamily. Different to the other tissue- specific PKC members, PKCδ is one of the widespread PKC isoform among several cells and tissues (Aris et al., 1993; Rahman et al., 2001; Kikkawa et al., 2002). Various reports indicate that activity of PKCδ is a critical player in cellular function to regulate cell fates, such as cell cycle, apoptosis or differentiation (Kikkawa et al., 2002; Vallee et al., 2004). Several reports indicate that PKCδ participates in the signal transduction of PYK2 (proline-rich tyrosine kinase 2) in response to the outside stimulation (Frank et al., 2002; Farshori et al., 2003). In addition, a phorbol ester activator of PKC, phorbol myristate acetate (PMA) has been demonstrated to stimulate the phosphorylations of PKCδ and PYK2 in pancreatic acinar cells (Wrenn, 2001). Both of the PYK2 and FAK (focal adhesion tyrosine kinases) have been shown to be the key signaling components to regulate cellular behaviors including migration, proliferation, and survival (Lipinski and Loftus, 2010; Lee and Yang, 2013). Several reports indicate that both of PKCδ (Woo et al., 2005; Yang S.F. et al., 2010; Mondrinos et al., 2014; Lee et al., 2015) and PYK2 (Zhao et al., 2014) affect ICAM-1 expression (Vallee et al., 2004). We suggested that PKCδ and PYK2 may be required for the S1P-induced VCAM-1 expression in HPAEpiCs.

In pulmonary inflammatory diseases, the accumulation of chronic inflammatory factors and imbalance between oxidant/antioxidant promote seriously cellular damages including the respiratory tissue injury. The ROS (reactive oxygen species) are considered to be the important mediators to affect cellular behaviors such as cell migration, adhesion, growth, differentiation, senescence, and apoptosis. The excessive amount of ROS generation was associated with respiratory inflammatory diseases (Lee and Yang, 2012). TNF-α, one of the pro-inflammatory mediators, activates the NADPH oxidase and leading to ROS overproduction which mediates the ICAM-1 and VCAM-1 inductions (Lee and Yang, 2012, 2013). Previous reports indicate that several responsible elements for transcription factors are identified to regulate ICAM-1 promoter, including NF-κB (Roebuck and Finnegan, 1999; Yang C.M. et al., 2010; Hadad et al., 2011). In inflammatory reactions, IKK-dependent IκB degradation is initiated to activate NF-κB. The released NF-κB subunit is translocated into nuclei and regulated the gene expressions (Baeuerle and Baltimore, 1996; Shih et al., 2015). Besides the typical regulated pathway, our findings indicated that NF-κB activity is also activated by another multiple mechanisms, such as PKCδ (Lin et al., 2014), PYK2 (Lee and Yang, 2013), and NADPH oxidase/ROS (Lee and Yang, 2013). These results demonstrate that various signaling molecules are involved in the regulation of ICAM-1 induction. However, the signal transductions participated in NF-κB-dependent ICAM-1 expression and monocyte adhesion on S1P-challenged HPAEpiCs remain unknown.

In addressing these questions, experiments were undertaken to investigate the effects of S1P on expression of ICAM-1 and monocyte adhesion on HPAEpiCs. These findings suggest that the increased expression of ICAM-1 and monocyte adhesion on S1P-challenged HPAEpiCs are mediated through Gq- and Gi/o-coupled receptor/PKCδ/PYK2/NADPH oxidase/ROS-dependent NF-κB activation. These findings proposed new insights of the mechanisms of S1P stimulation to regulate the expression of ICAM-1 on HPAEpiCs surface and thus expand the inflammation responses.

## Materials and Methods

### Materials

DMEM/F-12 medium, fetal bovine serum (FBS), TRIzol reagent and PLUS-Lipofectamine were from Invitrogen (Carsbad, CA, USA). Hybond C membrane and enhanced chemiluminescence (ECL) detection system were from GE Healthcare Biosciences (Buckinghamshire, UK). Anti-ICAM-1, anti-GAPDH, anti-PKCδ, anti-PYK2, anti-p47*^phox^*, and anti-p65 antibodies were from Santa Cruz (Santa Cruz, CA, USA). Anti-phospho-PKCδ, anti-phospho-PYK2, and anti-phospho-p65 antibodies were from Cell Signaling (Danvers, MA, USA). Actinomycin D (Act. D), cycloheximide (CHI), GP antagonist-2 (GPA2), GP antagonist-2A (GPA2A), Rottlerin, PF431396, Bay11-7082, Edaravone, diphenyleneiodonium chloride (DPI), and apocynin (APO) were from Biomol (Plymouth Meetings, PA, USA). S1P was from Cayman (Ann Arbor, MI, USA). Luciferase assay kit was from Promega (Madison, WI, USA). SDS-PAGE reagents were from MDBio Inc (Taipei, Taiwan). 2′,7′-dichlorodihydrofluorescein diacetate (DCFH-DA) and 2′,7′-Bis-(2-carboxyethyl)-5-(and-6)-carboxyfluorescein, acetoxymethyl ester (BCECF/AM) were from Molecular Probes (Eugene, OR, USA). All other reagents were from Sigma (St. Louis, MO, USA).

### Cell Culture and Treatment

Human pulmonary alveolar epithelial cells (HPAEpiCs) were purchased from the ScienCell Research Laboratories (San Diego, CA, USA) and grown as previously described (Lee et al., 2013a). Experiments were performed with cells from passages 4 to 8. Treatment of HPAEpiCs with DMSO or the pharmacological inhibitors alone had no significant effect on cell viability determined by a 2,3-bis-(2-methoxy-4-nitro-5-sulfophenyl)-2H-tetrazolium-5-carboxanilide (XTT) assay (data not shown).

### Transient Transfection with siRNAs

Human siRNAs of PKCδ (SASI_Hs01_00061170), PYK2 (SASI_Hs01_00032249), p47*^phox^* (SASI_Hs02_00302212), p65 (SASI_Hs01_00171091), or scrambled control (St. Louis, MO, USA) were used to knockdown the specific gene expression. The transient transfection of siRNA (100 nM) was performed with Metafectene transfection reagent according to the manufacturer’s protocol (Biontex, Germany).

### Preparation of Cell Extracts and Western Blot Analysis

Quiescent cells were treated with S1P for the indicated time intervals. The cells were harvested as previously described (Lee et al., 2013a). Samples were analyzed by using 10% SDS-PAGE and transferred to nitrocellulose membrane. Membranes were probed with an anti-ICAM-1 antibody (1:1000) and then membranes were incubated with horseradish peroxidase conjugated anti-rabbit antibody (1:2000) for 1 h at room temperature. The membranes were washed with tween-Tris buffered saline and detected by ECL reagents. The immunoblotting signals were captured by UVP BioSpectrum 500 Imaging System (Upland, CA, USA). The UN-SCAN-IT gel software (Orem, UT, USA) was used to quantify image densitometry.

### Total RNA Extraction and Real-time PCR Analysis

Total RNA were extracted with TRIzol reagent (Thermo Fisher, Waltham, MA, USA) according to the protocol of the manufacturer. The cDNA obtained from 5 μg total RNA was used to be a template for PCR amplification (Torre-Amione et al., 1996). Real-time PCR was performed with KAPA PROBE FAST ABI Prism^^®^^ qPCR kit (KK4705, Kapa Biosystems, Wilmington, MA, USA) and 7500 Real-Time PCR System (Applied Biosystems, Foster City, CA, USA) to analyze the amounts of ICAM-1 and GAPDH mRNA. Fold-changes of gene expression were calculated with the ΔΔCt method and all analysis were performed in triplicate (*n* = 3).

### Cell Adhesion Assay

Confluent HPAEpiCs on 6-well plates were treated with S1P for 16 h, and then adhesion assays were performed as previously described (Lin et al., 2016). Briefly, THP-1 cells (human acute monocytic leukemia cell line) were incubated with 10 μM BCECF/AM in RPMI-1640 medium (Gibco BRL, Grand Island, NY, USA) at 37°C for 1 h. HPAEpiCs were incubated with these labeled THP-1 cells (2 × 10^6^ cells/ml) for 1 h. The non-adherent THP-1 cells were removed by gently PBS-wash twice. The attached THP-1 cells were observed and measured with a fluorescence microscope (Zeiss, Axiovert 200 M). Experiments were performed in triplicate and repeated at least three times.

### Plasmid Construction, Transfection, and Luciferase Reporter Gene Assays

The human ICAM-1 (pIC-339) firefly luciferase was kindly provided by Dr. P. T. van der Saag (Hubrecht Laboratory, Utrecht, The Netherlands). All plasmids were prepared by using QIAGEN plasmid DNA preparation kits. ICAM-1-luc activity was determined using a luciferase assay system (Promega, Madison, WI, USA) as previously described (Lee et al., 2013b).

### Determination of NADPH Oxidase Activity by Chemiluminescence Assay

The Nox activity was examined by lucigenin chemiluminescence assay according to the previous report (Hsieh et al., 2012) with minor modification. After incubation, the collected cell pellet was resuspended with 35 μl of ice-cold RPMI-1640 medium on ice bath. To initiate the enzyme reaction, 5 μl of cell suspension (0.2 × 10^5^ cells) was added to 200 μl of pre-warmed (37°C) RPMI-1640 medium containing either NADPH (1 μM) or lucigenin (20 μM) and then the chemiluminescence was immediately measured by an Appliskan luminometer (Thermo^^®^^) in out-of-coincidence mode. Appropriate blanks and controls were established. Neither NADPH nor NADH enhanced the background chemiluminescence of lucigenin alone (30–40 counts per min). Chemiluminescence was continuously measured for 12 min, and the activity of NADPH oxidase was expressed as counts per million cells.

### Measurement of Intracellular ROS Generation

The measurement of generation of intracellular ROS was performed with peroxide-sensitive fluorescent probe (2′,7′-dichlorofluorescein diacetate, DCF-DA) as previous described (Lin et al., 2016). Washed HPAEpiCs were labeled with 10 μM DCFH-DA in HBSS for 30 min. Subsequently, the free DCFH-DA was removed and replaced with fresh medium. HPAEpiCs were treated with various concentrations of S1P. Cells were detached with trypsin/EDTA, and the fluorescence intensity of the cells was analyzed with FACScan flow cytometer (BD Biosciences, San Jose, CA, USA) at 495 nm excitation and 529 nm emission for DCF.

### Immunofluorescence Staining

Sphingosine-1-phosphate-treated HPAEpiCs for the indicated time intervals were washed twice with ice-cold PBS and fixed with 4% paraformaldehyde. The fixed cells were permeabilized, and probed with the primary antibody, anti-p65 antibody, as previously described (Lee et al., 2013b). The images were observed and captured with fluorescence microscope (Zeiss, Axiovert 200 M).

### Chromatin Immunoprecipitation Assay

The chromatin immunoprecipitation (ChIP) analysis was performed to analysis the transcription factor association on human ICAM-1 promoter *in vivo* as previously described (Lee et al., 2007). Briefly, HPAEpiCs were cross-linked with 1% formaldehyde at 37°C for 10 min and stop this reaction with 0.125 M glycin. ChIP assay kit (Upstate) was used to prepare the soluble chromatin/protein complex according to the manufacturer’s protocol and immunoprecipitated with anti-p65 antibody or normal IgG. Following washes and elution, immunoprecipitated complex was heated overnight at 65°C to remove cross-linking of DNA and protein. DNA fragments were purified by phenol-chloroform extraction and ethanol precipitation. PCR fragments were analyzed on 2% agarose in 1X TAE gel containing ethidium bromide.

### Animal Care and Experimental Procedures

Male ICR mice aged 6–8 weeks were purchased from the National Laboratory Animal Centre (Taipei, Taiwan) and handled according to the guidelines of Animal Care Committee of Chang Gung University and NIH Guides for the Care and Use of Laboratory Animals. ICR mice were anesthetized with Zoletil (40 mg/kg) and placed individually on a board in a near vertical position and the tongues were withdrawn with a lined forceps. S1P (3 mg/kg body weight, 50 μl of 5 mM S1P, about 30 g of body weight/mouse) was placed posterior in the throat and aspirated into lungs. DMSO- administrated mice were used to be the control group. After 15 min, regained consciousness mice were i.p. given one dose of GPA2A, Rottlerin, PF431396, DPI, APO, Edaravone, or Bay11-7082 (2 mg/kg) for 2 h prior to S1P treatment, and sacrificed after 24 h. To examine the levels of ICAM-1 mRNA and protein expression, lung tissues were collected, homogenized and subjected to real-time PCR and Western blot, as previously described (Lee et al., 2007).

### Isolation of Bronchoalveolar Lavage (BAL) Fluid

Mice were injected with 3 mg/kg S1P for 24 h and then killed to harvest BAL fluid. BAL fluid was obtained through tracheal cannula using aliquots of 1 ml ice–cold PBS, as previously described (Lee et al., 2007). BAL fluid was collected and centrifuged at 500 × *g* at 4°C, and cell pellets were washed and re-suspended in PBS. The number of leukocyte was measured with hemocytometer.

### Statistical Analysis of Data

All the data were expressed as the mean or mean ± SEM of three individual experiments performed in duplicate or triplicate, as previously described (Lin et al., 2016). The significance of difference between two groups was determined by paired two-tailed Student’s *t*-test for western blot data. All others statistical analyses are comparison of multiple groups, a GraphPad Prism Program (GraphPad, San Diego, CA, USA) by one-way analysis of variance (ANOVA) followed with Tukey’s *post hoc* test has been used. A *P* < 0.05 value was considered significant.

## Results

### S1P-Induced ICAM-1 Expression in Transcription and Translation

**FIGURE 1:**
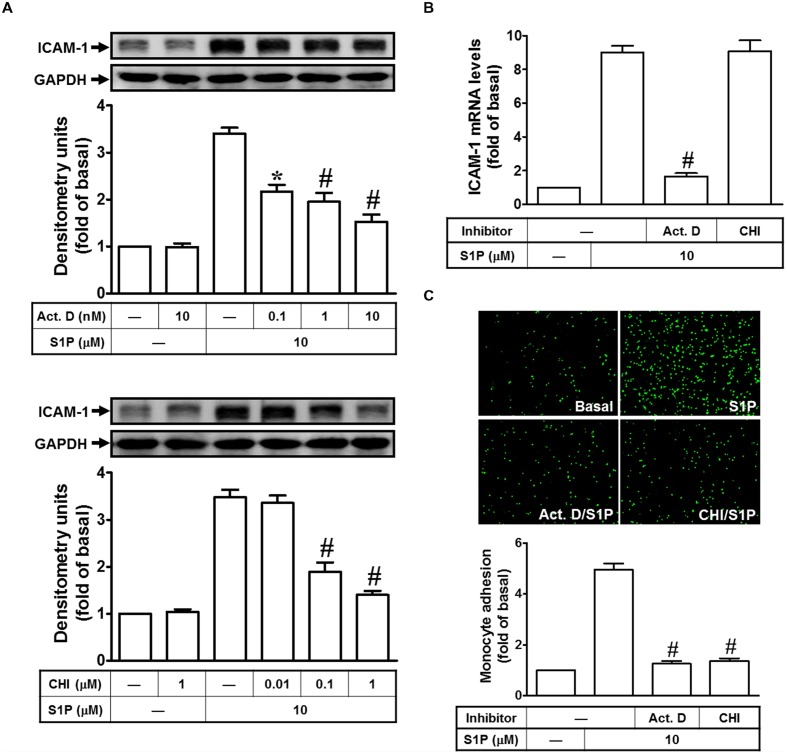
**Sphingosine-1-phosphate-induced ICAM-1 expression requires ongoing transcription and translation. (A)** Cells were pretreated with Actinomycin D (Act. D) or CHI for 1 h, and then incubated with 10 μM S1P for 16 h. The ICAM-1 protein expression was determined by Western blot. **(B)** Cells were pretreated with Act. D (10 nM) or CHI (1 μM) for 1 h, and then incubated with 10 μM S1P for 4 h. The ICAM-1 mRNA expression was determined by real-time PCR. **(C)** Cells were pretreated with Act. D (10 nM) or CHI (1 μM) for 1 h, and then incubated with 10 μM S1P for 16 h. The THP-1 cells adherence was measured. Data are expressed as mean ± SEM of three independent experiments. ^∗^*P* < 0.05; ^#^*P* < 0.01, as compared with the cells exposed to S1P alone.

Our previous report indicated that the concentration of S1P (10 μM) used to obtain a maximal ICAM-1 expression which was applied in this study (Lin et al., 2015). To examine the mechanisms of S1P-induced ICAM-1 expression, HPAEpiCs were stimulated with S1P (10 μM) in the pretreatment with (or without) (Act. D, transcriptional inhibitor) or cycloheximide (CHI, translational inhibitor) and then Western blotting was performed to analyze ICAM-1 protein expression. Our data indicated that both of Act. D and CHI treatments can attenuate the S1P-mediated ICAM-1 induction in a dose-dependent manner (**Figure 1A**). Besides, Act. D, but not CHI, significantly reduced S1P-induced ICAM-1 mRNA expression in HPAEpiCs (**Figure 1B**). Next, we found that adhesion of THP-1 to HPAEpiCs was enhanced by S1P challenge about fivefold, which was also obviously inhibited by pretreatment with Act. D or CHI (**Figure 1C**). Taken together, we suggested that S1P-induced ICAM-1 expression depends on *de novo* protein synthesis in HPAEpiCs.

### Gq- and Gi/o-Coupled Receptors Play Key Roles in S1P-Induced ICAM-1 Expression

**FIGURE 2:**
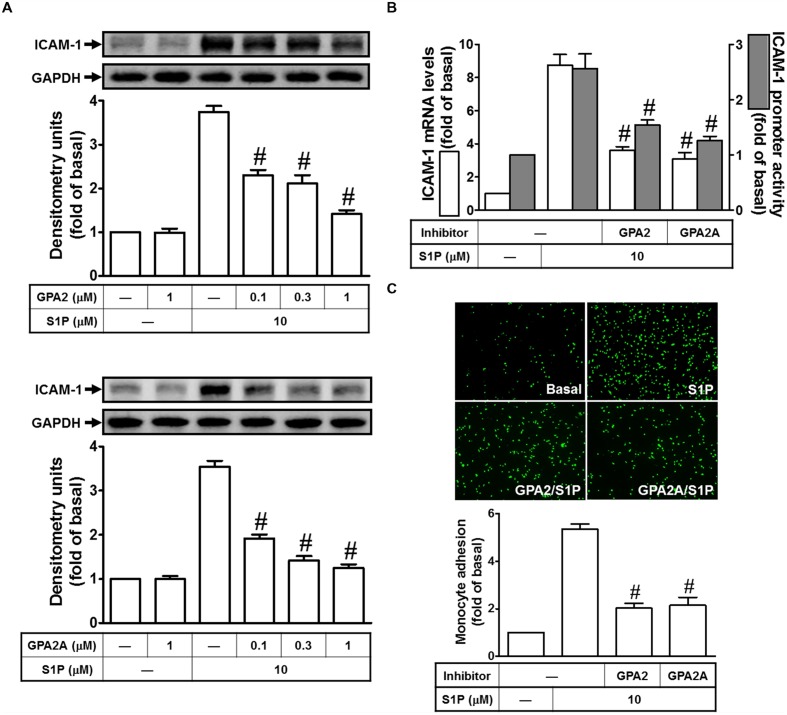
**Gq-coupled receptor and Gi/o-coupled receptor play key roles in S1P-induced ICAM-1 expression. (A)** Cells were pretreated with GPA2 or GPA2A for 1 h, and then incubated with 10 μM S1P for 16 h. The ICAM-1 protein expression was determined by Western blot. **(B)** Cells were pretreated with GPA2 (1 μM) or GPA2A (1 μM) for 1 h, and then incubated with 10 μM S1P for 4 h. The ICAM-1 mRNA expression and promoter activity were determined by real-time PCR and promoter assay, respectively. **(C)** Cells were pretreated with GPA2 (1 μM) or GPA2A (1 μM) for 1 h, and then incubated with 10 μM S1P for 16 h. The THP-1 cells adherence was measured. Data are expressed as mean ± SEM of three independent experiments. ^#^*P* < 0.01, as compared with the cells exposed to S1P alone.

Sphingosine-1-phosphate regulates numerous cellular responses, including formation of adherent junctions, proliferation, migration, and survival (Ryan and Spiegel, 2008; Takashima et al., 2008; Li et al., 2009). These myriad effects are partly elicited by binding of S1P to a family of five G protein–coupled receptors, termed S1PR1–5. S1PR1, S1PR2, and S1PR3 are ubiquitously expressed, whereas the levels of S1PR4 and S1PR5 expression are predominantly existed in immune cells, CNS, and some organs. Indeed, S1PR1, 2 and 3 are expressed on HPAEpiCs (Lin et al., 2015). Thus, we further investigated whether Gq- or Gi/o-coupled receptor was involved in S1P-induced ICAM-1 expression. As shown in **Figures 2A,B**, our data indicated that pretreatments of GPA2A (Gq-coupled receptor antagonist) and GPA2 (Gi/o-coupled receptor antagonist) concentration-dependently block the S1P-induced ICAM-1 expression in protein (∼63%) and mRNA (∼68%), respectively. The analysis of promoter activity (∼62%) also exhibit consistent results. Finally, we observed that S1P-enhanced monocyte adhesion was also reduced to approximate 70% of control by these two inhibitors (**Figure 2C**). Taken together, we suggested that Gq- and Gi/o-coupled receptors play key roles in S1P-induced ICAM-1 expression in HPAEpiCs.

### PKCδ is Involved in S1P-Induced ICAM-1 Expression

**FIGURE 3:**
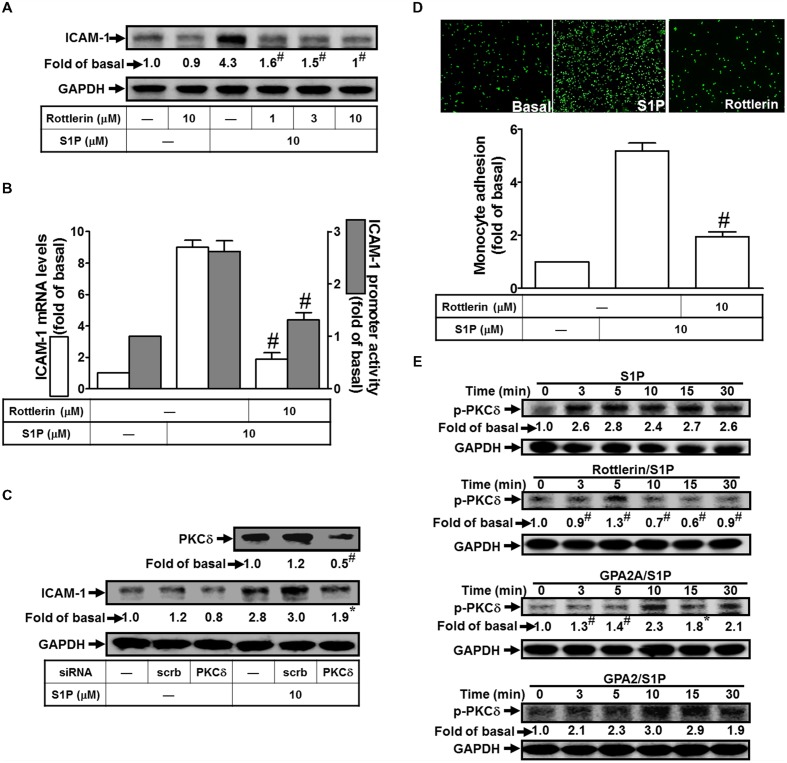
**PKCδ is involved in S1P-induced ICAM-1 expression. (A)** Cells were pretreated with Rottlerin for 1 h, and then incubated with 10 μM S1P for 16 h. The ICAM-1 protein expression was determined by Western blot. **(B)** Cells were pretreated with Rottlerin (10 μM) for 1 h, and then incubated with 10 μM S1P for 4 h. The ICAM-1 mRNA expression and promoter activity were determined by real-time PCR and promoter assay, respectively. **(C)** Cells were transfected with siRNA of scrambled or PKCδ, and then incubated with 10 μM S1P for 16 h. The levels of PKCδ and ICAM-1 proteins were determined by Western blot. **(D)** Cells were pretreated with Rottlerin (10 μM) for 1 h, and then incubated with 10 μM S1P for 16 h. The THP-1 cells adherence was measured. **(E)** Cells were pretreated without or with Rottlerin, GPA2A, or GPA2 for 1 h, and then incubated with 10 μM S1P for the indicated time intervals. The levels of phospho-PKCδ were determined by Western blot. Data are expressed as mean ± SEM of three independent experiments. ^#^*P* < 0.01, as compared with the cells exposed to S1P alone.

Several lines of evidence indicate that PKCδ activity plays critical roles to regulate cellular physiological functions (Kikkawa et al., 2002). We also obtained the similar results that pretreatment of Rottlerin (selective PKCδ inhibitor) significantly attenuate S1P-induced ICAM-1 protein expression by approximate 85% (**Figure 3A**). This inhibitory phenomenon was also exhibited in mRNA by approximate 95% (**Figure 3B**) and promoter activity by approximate 55% (**Figure 3B**). To confirm that PKCδ is involved in S1P-induced ICAM-1 expression, siRNA transfection was used to knockdown PKCδ expression. The results of Western blotting indicated that PKCδ siRNA transfection reduces PKCδ protein by approximate 55%, and then inhibits the S1P-induced ICAM-1 expression by approximate 40% (**Figure 3C**). Consistently, pretreatment with Rottlerin also decreased monocyte adhesion to the cell surface of HPAEpiCs challenged with S1P by approximate 65% (**Figure 3D**). Especially, we found that S1P can trigger PKCδ phosphorylation with a time-dependent manner, which was reduced by pretreatment of Rottlerin and GPA2A, but not GPA2 (**Figure 3E**). Taken together, we demonstrated that the Gq-coupled receptor/PKCδ activation mediate S1P stimulated ICAM-1 expression in these cells.

### PYK2 is a Key Point in Regulating S1P-Induced ICAM-1 Expression

**FIGURE 4:**
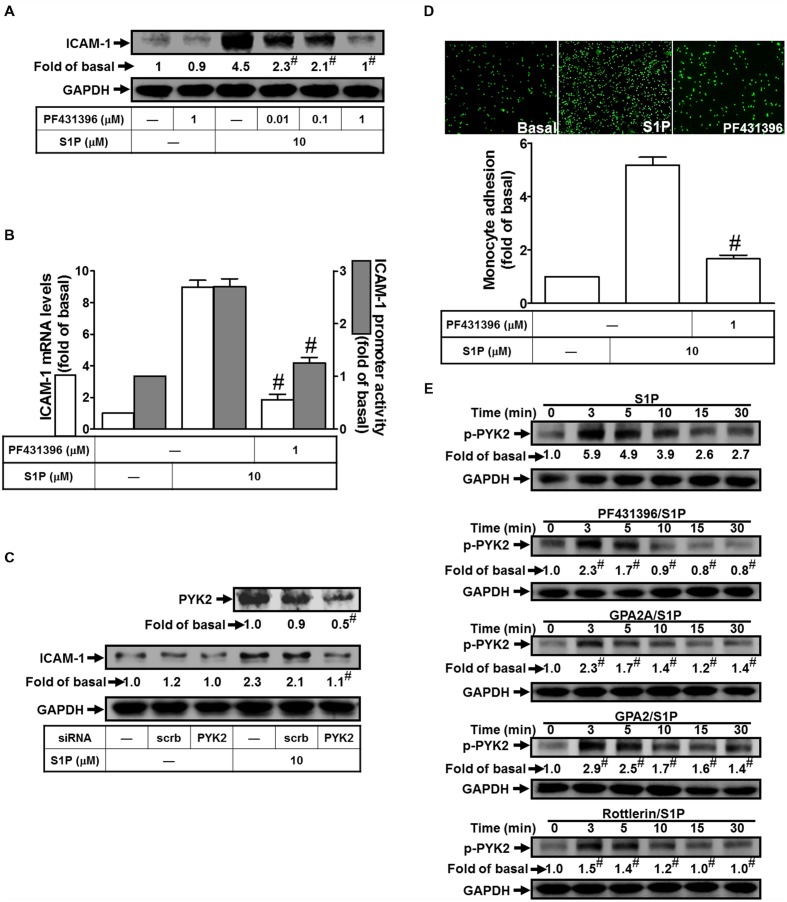
**PYK2 plays a key role in regulating S1P-induced ICAM-1 expression. (A)** Cells were pretreated with PF431396 for 1 h, and then incubated with 10 μM S1P for 16 h. The ICAM-1 protein expression was determined by Western blot. **(B)** Cells were pretreated with PF431396 (1 μM) for 1 h, and then incubated with 10 μM S1P for 4 h. The ICAM-1 mRNA expression and promoter activity were determined by real-time PCR and promoter assay, respectively. **(C)** Cells were transfected with siRNA of scrambled or PYK2, and then incubated with 10 μM S1P for 16 h. The levels of PYK2 and ICAM-1 proteins were determined by Western blot. **(D)** Cells were pretreated with PF431396 (1 μM) for 1 h, and then incubated with 10 μM S1P for 16 h. The THP-1 cells adherence was measured. **(E)** Cells were pretreated without or with PF431396, Rottlerin, GPA2A, or GPA2 for 1 h, and then incubated with 10 μM S1P for the indicated time intervals. The levels of phospho-PYK2 were determined by Western blot. Data are expressed as mean ± SEM of three independent experiments. ^#^*P* < 0.01, as compared with the cells exposed to S1P alone.

Exposure of PMA has been shown to stimulate the phosphorylation of PKCδ and PYK2 in pancreas acinar cells (Wrenn, 2001). Based on this finding, we focused to study the role of PYK2 in S1P-induced ICAM-1 expression. The results of Western blotting indicated that pretreatment of PF431396 (an inhibitor of PYK2) reduced S1P-induced ICAM-1 protein expression by approximate 91% (**Figure 4A**). The mRNA level and promoter activity of ICAM-1 also exhibited consistent inhibitory results by approximate 89 and 55%, respectively (**Figure 4B**). To investigate the regulatory role of PYK2 in the pathway of S1P-induced ICAM-1 expression, PYK2 siRNA was used to block the PYK2 expression. As expected, PYK2 siRNA transfection reduced the PYK2 total protein about 50%, and then significantly attenuated S1P-induced ICAM-1 expression from 2.1-fold to 1.1-fold (**Figure 4C**). Moreover, blocking the activity of PYK2 with PF431396 also reduced monocyte adhesion to HPAEpiCs challenged with S1P by approximate 65% (**Figure 4D**). Finally, we found that 10 μM S1P time-dependently stimulated the phosphorylation of PYK2 which was attenuated by PF431396, GPA2A, GPA2, and Rottlerin (**Figure 4E**). Taken together, we suggested that S1P-activated PKCδ and PYK2 phosphorylation results in ICAM-1 expression in HPAEpiCs.

### NADPH Oxidase-Dependent ROS Generation is Participated in S1P-Induced ICAM-1 Expression

**FIGURE 5:**
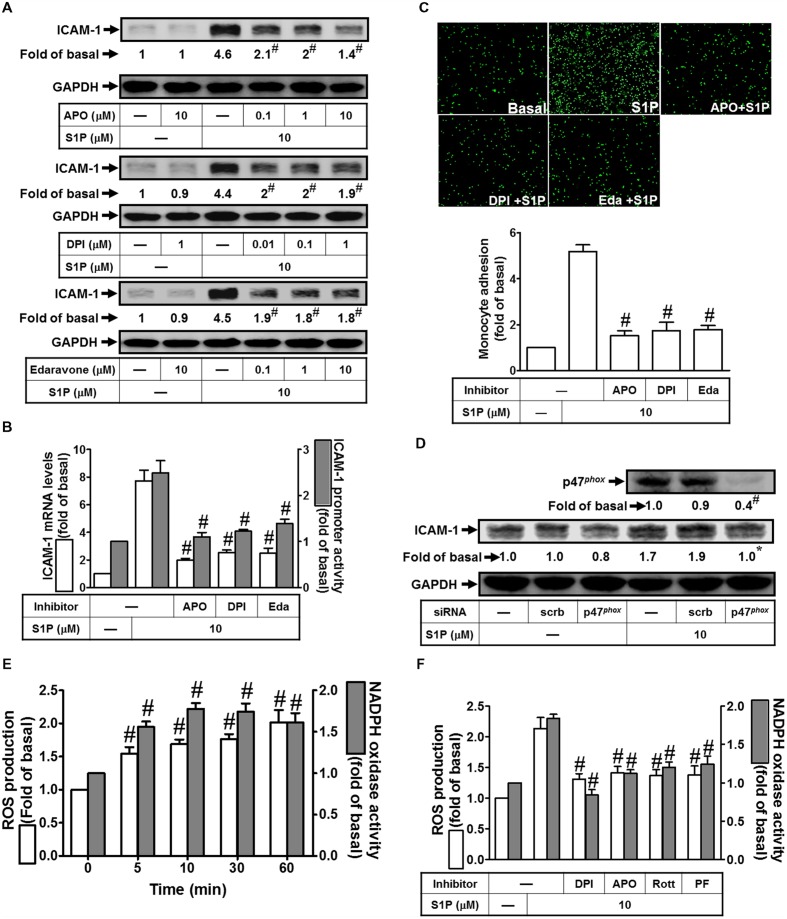
**NADPH oxidase/ROS play key roles in mediating S1P-induced ICAM-1 expression. (A)** Cells were pretreated with APO, DPI, or Edaravone for 1 h, and then incubated with 10 μM S1P for 16 h. The ICAM-1 protein expression was determined by Western blot. **(B)** Cells were pretreated with APO (10 μM), DPI (1 μM), or Edaravone (10 μM) for 1 h, and then incubated with 10 μM S1P for 4 h. The ICAM-1 mRNA expression and promoter activity were determined by real-time PCR and promoter assay, respectively. **(C)** Cells were pretreated with APO (10 μM), DPI (1 μM), or Edaravone (10 μM) for 1 h, and then incubated with 10 μM S1P for 16 h. The THP-1 cells adherence was measured. **(D)** Cells were transfected with siRNA of scrambled or p47*^phox^*, and then incubated with 10 μM S1P for 16 h. The levels of p47*^phox^* and ICAM-1 proteins were determined by Western blot. **(E)** Cells were treated with 10 μM S1P for the indicated times. The ROS production and NADPH oxidase activity were measured. **(F)** Cells were pretreated with DPI, APO, Rottlerin, or PF431396 for 1 h, and then incubated with 10 μM S1P for 60 min or 10 min. The ROS production and NADPH oxidase activity were measured. Data are expressed as mean ± SEM of three independent experiments. ^#^*P* < 0.01, as compared with the cells exposed to S1P alone **(A–C,F)** or vehicle alone **(E)**.

Excessive production of ROS by NADPH oxidase from tissue injury is associated with a range of respiratory inflammatory diseases (Lee and Yang, 2012). Our previous studies also indicated that ICAM-1 induction by TNF-α-treatment is mediated by the NADPH oxidase activation and ROS overproduction (Lee and Yang, 2012). Moreover, our data indicated that all of the inhibitors of NADPH oxidase (DPI and APO) and ROS (Edaravone) markedly reduced S1P-induced ICAM-1 protein (∼70, 57, and 60%) and mRNA levels (∼74, 67, and 66%), as well as promoter activity (∼55, 50, and 41%) (**Figures 5A,B**). In addition, these three inhibitors also reduced monocyte adhesion to HPAEpiCs challenged with 10 μM S1P (**Figure 5C**). Activated NADPH oxidase is dependent on a multimeric protein complex formation, in which consists with at least three cytosolic subunits (p47*^phox^*, p67*^phox^*, and p40*^phox^*) interaction. Because conformational change of phosphorylated p47*^phox^* associated with p22*^phox^*, p47*^phox^* plays a role as an “organizer subunit” to organize other cytosolic factor translocations (Lee and Yang, 2012). We further examined the role of p47*^phox^* in S1P-induced ICAM-1 expression. As shown in **Figure 5D**, p47*^phox^* siRNA transfection knocked down the total p47*^phox^* protein by approximate 60%, and then inhibited S1P-enhanced ICAM-1 expression by approximate 41%. Moreover, S1P time-dependently induced both of the NADPH oxidase activity and intracellular ROS generation (**Figure 5E**). Consistently, activation of NADPH oxidase activity and ROS generation were inhibited by DPI, APO, Rottlerin, or PF431396 (**Figure 5F**). Taken together, we suggested that S1P induces NADPH oxidase/ROS-dependent ICAM-1 expression in HPAEpiCs.

### NF-κB Plays a Key Role in S1P-Induced ICAM-1 Expression and Monocyte Adhesion

**FIGURE 6:**
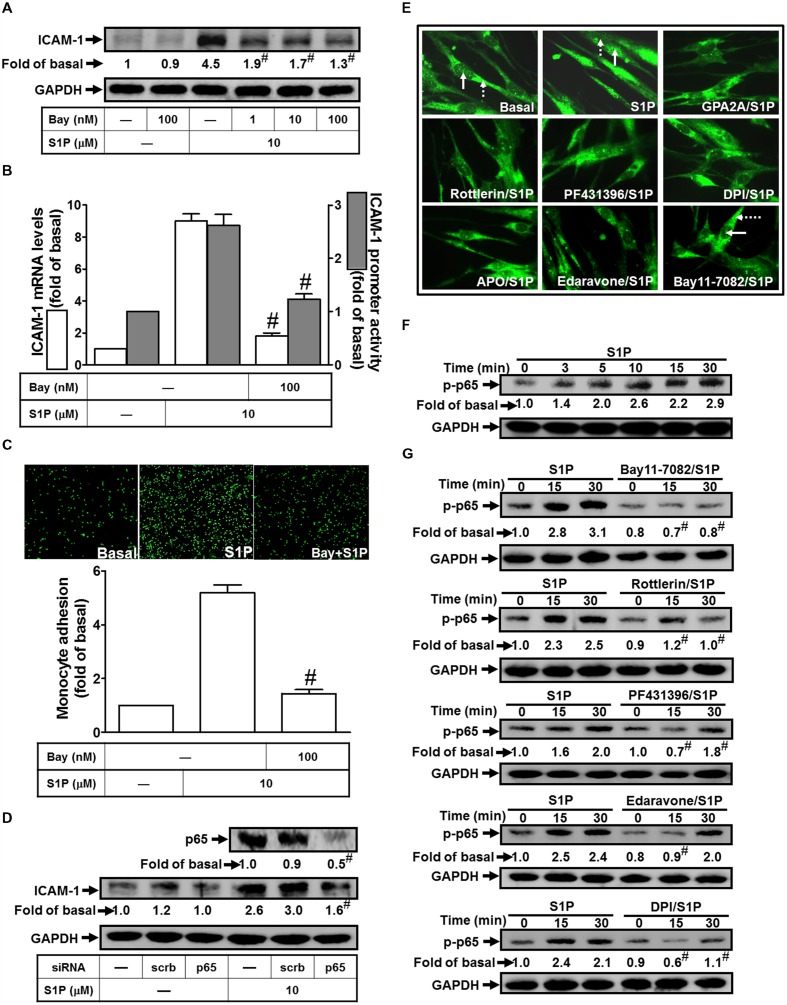
**NF-κB plays a key role in mediating S1P-induced ICAM-1 expression. (A)** Cells were pretreated with Bay11-7082 for 1 h, and then incubated with 10 μM S1P for 16 h. The ICAM-1 protein expression was determined by Western blot. **(B)** Cells were pretreated with Bay11-7082 (100 nM) for 1 h, and then incubated with 10 μM S1P for 4 h. The ICAM-1 mRNA expression and promoter activity were determined by real-time PCR and promoter assay, respectively. **(C)** Cells were pretreated with Bay11-7082 (100 nM) for 1 h, and then incubated with 10 μM S1P for 16 h. The THP-1 cells adherence was measured. **(D)** Cells were transfected with siRNA of scrambled or p65, and then incubated with 10 μM S1P for 16 h. The levels of p65 and ICAM-1 proteins were determined by Western blot. **(E)** Cells were pretreated with GPA2A, Rottlerin, PF431396, DPI, APO, Edaravone, Bay11-7082 for 1 h or the vehicle control (Basal), and then incubated with 10 μM S1P for 30 min. Cells were fixed and then labeled with an anti-p65 antibody and then FITC-conjugated secondary antibody. Individual cells were enlarged with 1000x and imaged. The nuclear regions are indicated with solid arrows and the cytoplasmic regions are indicated with dashed arrows. **(F)** Cells were treated with 10 μM S1P for the indicated times or **(G)** pretreated with Bay11-7082, Rottlerin, PF431396, Edaravone, or DPI for 1 h, and then incubated with 10 μM S1P for the indicated times. The levels of phospho-p65 were determined by Western blot. Data are expressed as mean ± SEM of three independent experiments. ^#^*P* < 0.01, as compared with the cells exposed to S1P alone.

Activation of the NF-κB is dependent on the phosphorylation and degradation of IκB (Baeuerle and Baltimore, 1996; Shih et al., 2015). Upon degradation of IκB, the NF-κB complex translocates into the nucleus to turn on the transcription of specific genes that have the κB sites in their promoter regions. To examine whether NF-κB participates in S1P-imediated ICAM-1 expression, Bay11-7082 (an inhibitor of NF-κB) was used to study VCAM-1 expression in HPAEpiCs. We found that pretreatment with Bay11-7082 significantly reduced S1P-induced ICAM-1 protein in a dose-dependent manner, by approximate 71% (**Figure 6A**). We also obtained the consistent results at transcriptional analysis that pretreatment of Bay11-7082 attenuates both of the S1P-induced VCAM-1 mRNA expression approximate 71% and promoter activity about 77%, respectively (**Figure 6B**). Importantly, Bay11-7082 also attenuated monocyte adhesion to S1P-challenged HPAEpiCs (**Figure 6C**). To further confirm that NF-κB is a targeted transcription factor in S1P-induced ICAM-1 expression in HPAEpiCs, transfection of p65 siRNA was used to clarify the role of NF-κB in the S1P-mediated response. The results of Western blotting indicated that transfection with p65 siRNA significantly reduced p65 protein expression by approximate 51%, and then blocked S1P-induced ICAM-1 expression from 3.0-fold to 1.6-fold in HPAEpiCs (**Figure 6D**). Moreover, in our study, we showed that S1P markedly induced NF-κB 65 nuclear translocation, which was inhibited by GPA2A, Rottlerin, PF431396, DPI, APO, Edaravone, or Bay11-7082 (**Figure 6E**). On the other hand, we also found that 10 μM S1P stimulated NF-κB p65 phosphorylation in a time-dependent manner (**Figure 6F**, within 30 min). This induction was also reduced by pretreatment with Bay11-7082, Rottlerin, PF431396, Edaravone, and DPI (**Figure 6G**). Based on these results, we demonstrated that S1P initiates ROS-dependent NF-κB pathway to induce ICAM-1 expression on HPAEpiCs and resulted in the monocyte attachment.

### S1P Induces ICAM-1 Expression in Lung Fractions of Mice

**FIGURE 7:**
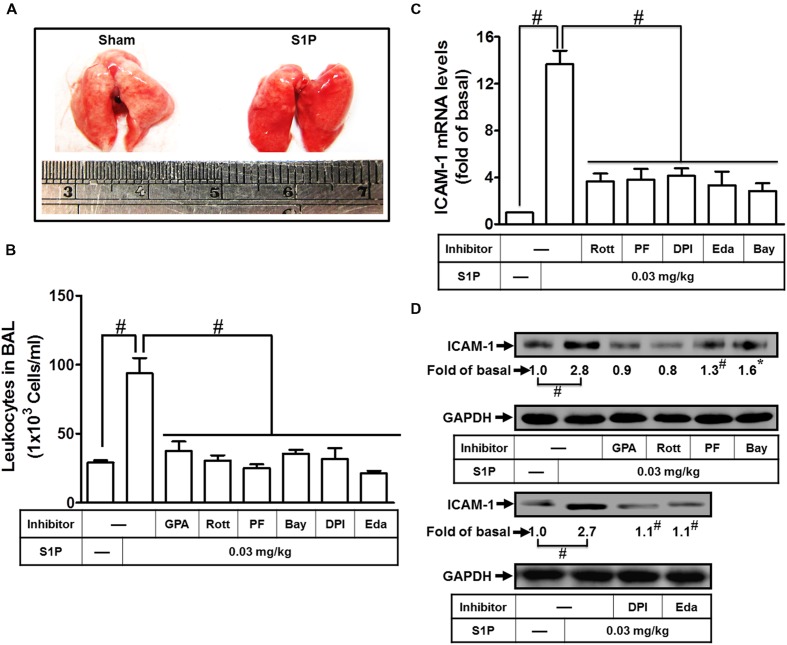
**Sphingosine-1-phosphate induces ICAM-1 expression in lung fractions of mice. (A)** Mice were intratracheally injected with S1P (3 mg/kg) for 24 h, and then the appearances of the lungs were observed. Mice were intratracheally injected with S1P (3 mg/kg) for 24 h. **(B–D)** Mice were i.p. given one dose of DMSO, Rottlerin, PF431396, DPI, Edaravone, Bay11-7082, or GPA2A (2 mg/kg) for 2 h before S1P treatment, and then sacrificed after 24 h. **(B)** BAL fluid was acquired and leukocyte count was determined by a hemocytometer. Lung tissues were homogenized to extract proteins and mRNA. The levels of ICAM-1 mRNA **(C)** and protein **(D)** were determined by real-time PCR and Western blot, respectively. Data are expressed as mean ± SEM of three independent experiments (*n* = 3). ^#^*P* < 0.01, as compared with the mice intratracheally injected with S1P alone.

To analyze the function of S1P, mice were intratracheally administered with S1P. The phenotype of lung indicated that S1P markedly caused pulmonary hematoma (**Figure 7A**). To further analyze the effects of pharmacological inhibitors on S1P-treated mice, mice were i.p. administered with Rottlerin, PF431396, DPI, Edaravone, Bay11-7082, or GPA2A for 2 h, and then followed with (3 mg/kg) S1P. The number of leukocytes (eosinophils and neutrophils) also indicated that after S1P treatment, leukocyte number is significantly elevated in BAL fluid and reduced by these inhibitors (**Figure 7B**). Besides, the total RNA and protein were extracted from lung tissues to analyze the levels of ICAM-1 expression. The results of RT/Real-time PCR indicated that all of the inhibitors Rottlerin, PF431396, DPI, Edaravone, and Bay11-7082 significantly attenuated S1P-induced ICAM-1 mRNA by approximate 71% (**Figure 7C**). And the results of Western blotting also indicated that GPA2A, Rottlerin, PF431396, Bay11-7082, DPI, and Edaravone significantly inhibited S1P-induced ICAM-1 protein expression by approximate 64% in lung fractions of mice (**Figure 7D**). These data demonstrated that S1P-induced lung inflammation is, at least in part, mediated through an ICAM-1-dependent manner associated with leukocyte recruitment in the lungs.

## Discussion

**FIGURE 8:**
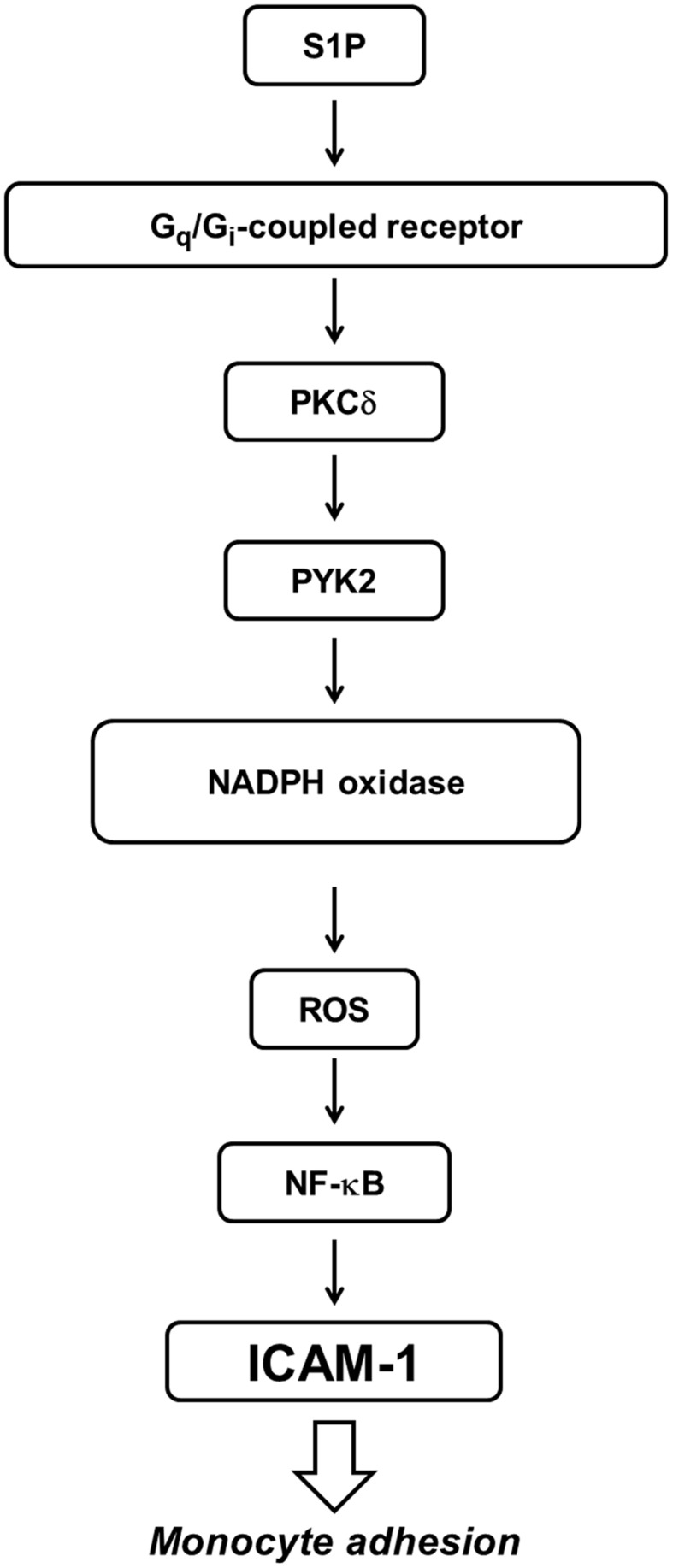
**Proposed model to illustrate the signaling pathways involved in ICAM-1 expression and monocyte adhesion in HPAEpiCs challenged with S1P.** S1P-induced ICAM-1 expression and monocyte adhesion is mediated through Gq-/Gi-coupled receptor/PKCδ/PYK2/NADPH oxidase/ROS-dependent NF-κB activation.

Asthma, COPD, and several chronic pulmonary diseases are characterized by various extents of continuous inflammation and airway remodeling in the respiratory system. The bioactive sphingolipid metabolite, S1P, activates EDG (endothelial differentiation gene) receptors and promotes the pro-inflammatory cytokine expression to enhance allergic responses in asthma (Ammit et al., 2001). Moreover, stimulation of S1P promotes expression of cell surface adhesion molecules to accelerate lung inflammatory injury. Previous reports indicated that S1P concentration in plasma and tissue exhibits different levels according to various experimental conditions (Ammit et al., 2001; Yatomi, 2008; Price et al., 2013). The S1P concentrations have been shown to be elevated in pulmonary patients as compared with normal subjects (Ammit et al., 2001; Knapp et al., 2009, 2013). However, the molecular mechanisms of S1P induced ICAM-1 expression and infiltration of monocytes are not fully defined in HPAEpiCs. In contrast to the human, the S1P concentration is about 0.2–1 μM in the plasma of mice (Hisano et al., 2012; Kowalski et al., 2013). In this study and our previous data (Lin et al., 2015), 10 μM S1P was used to obtain the maximal responses and this induction can be manipulated with other approached methods such as the inhibitors treatment and siRNA transfection to dissect out the regulation of signal transduction pathways. The concentrations of inhibitors used were about 30-folds of their IC_50_ values in the literatures which allowed us to investigate the roles of signaling molecules involved in S1P-mediated responses in HPAEpiCs. The present studies demonstrated that S1P-induced ICAM-1 expression was, at least in part, mediated via Gq- and Gi/o-coupled receptors/PKCδ/PYK2/NADPH oxidase/ROS-dependent NF-κB activation (**Figure 8**). Genetic silencing through transfection with siRNA of PKCδ, PYK2, p47*^phox^*, or p65 and pretreatment with the inhibitor of PKCδ (Rottlerin), PYK2 (PF431396), NADPH oxidase (DPI or APO), ROS (Edaravone), or NF-κB (Bay11-7082) abrogated S1P-induced ICAM-1 expression and monocyte adhesion. Therefore, activation of S1P receptors by S1P causes inflammatory responses through ICAM-1 up-regulation.

Several lines of evidence have reported that diverse biological effects of S1P are mediated through S1PRs-dependent activity (Li et al., 2009; Aarthi et al., 2011). Moreover, several reports identify that S1PR1, S1PR2, and S1PR3 express on various cell types (Ishii et al., 2004; Brinkmann, 2007; Takashima et al., 2008; Lin et al., 2015). Indeed, we also found that all of these three receptors (S1PR1–3) are expressed on HPAEpiCs to mediate S1P-responses (Lin et al., 2015). S1P receptors belong to the member of G protein–coupled receptors. The interaction between G protein–coupled receptors and G protein plays an import role in signal transmission to mediate cellular responses and behaviors (Shen et al., 2013, 2015). We found that, in HPAEpiCs, both of the Gq- and Gi/o-coupled receptors are involved in S1P-induced ICAM-1 expression and monocyte adhesion on HPAEpiCs (**Figure 2**).

In the PKC family, at least 11 phospholipid-dependent Ser/Thr kinases, are discovered and engage in a wide array of pathways to regulate cellular responses and behaviors. According to the cofactors, PKC isoforms are classified into three categories dependent on the requirement of optimal phospholipid-dependent catalytic activity (Lee and Yang, 2013). S1P has been shown to cause PKCδ activation in A549 human lung adenocarcinoma cells (Meacci et al., 2003) or human-airway epithelial cells (Ghelli et al., 2002). Sobel et al. (2013) indicate that agonists of S1P receptor can activate both of S1P2 and S1P3 receptors to mediate pro-fibrotic responses in human lung fibroblasts. Previous report also indicates that Gq-linked PKCδ promotes the downstream cellular reactions (Tu et al., 2007). Interestingly, we noticed that only Gq-coupled receptors can be linked to activate S1P-dependent PKCδ activation in HPAEpiCs but not Gi/o-coupled receptor. This is consistent to our observations that blocking the activated pathway of PKCδ effectively reduced S1P-induced ICAM-1 expression and monocyte adhesion.

Exposure to various stimuli could regulate cell migration, proliferation, and cell survival which may be mediated through the focal adhesion tyrosine kinases FAK and PYK2 acted as critical mediators in these responses (Lee and Yang, 2013). PYK2 is activated by a variety of GPCRs and inflammatory cytokines that elevate intracellular calcium concentrations (Li et al., 2014). In HPAEpiCs, we also demonstrated that S1P mediated ICAM-1 expression and monocyte adhesion via PYK2 activation. Exposure of acinar cells to PMA has been shown to result in increased phosphorylation of PKCδ and PYK2 (Wrenn, 2001). In our study, we focused on the inhibition of PKCδ reduced S1P-mediated PYK2 phosphorylation. Indeed, we found that both of the inhibitors of Gq-coupled receptor (GPA2A) and Gi/o-coupled receptor (GPA2) can attenuate the S1P-induced ICAM-1 expression (**Figure 2**) and PYK2 phosphorylation (**Figure 4E**). It is worth noting that pretreatment with rottlerin attenuated PYK2 phosphorylation stimulated by S1P, indicating that PYK2 is a downstream component of PKCδ. However, to analyze the phosphorylation of PKCδ (**Figure 3E**), we noticed that Gq-coupled receptor mainly plays a key role in this response, but not Gi/o-coupled receptor. Thus, PKCδ-activated PYK2 plays important roles in regulating S1P-triggered pulmonary diseases.

Cells and tissues are routinely subjected to sublethal doses of various oxidants, either exogenously through environmental exposure or endogenously through inflammatory processes (Lee and Yang, 2012). ROS are intracellularly generated from several sources, including mitochondrial respiration, cytochrome P450, the NADPH oxidase, xanthine oxidase, and arachidonic acid metabolism (Lee and Yang, 2012). Harijith et al. (2013) indicate that exogenous S1P stimulates intracellular ROS generation in human lung microvascular endothelial cells. Golan et al. (2012) also indicate that S1P can activate S1PR1-mediated ROS signaling and SDF-1 release to promote murine progenitor cell egress and mobilization. In NIH3T3 fibroblasts, S1P treatment can activate the NADPH oxidase and upregulate the generation of intracellular H_2_O_2_ (Catarzi et al., 2011). Moreover, we obtained the data showing that an important linkage of NADPH oxidase is established in S1P-induced ROS generation and leading to ICAM-1 in HPAEpiCs. In the future, other sources, such as xanthine oxidase or mitochondrial respiration are needed investigating whether S1P can regulate these ROS generation processes. PKCδ plays a role in the regulation of NADPH oxidase 1-mediated oxidative stress (Cristovao et al., 2013). PYK2 has also been shown to regulate ROS generation (Katsume et al., 2011). In our study, we established that S1P induced NADPH oxidase activation and ROS production via a PKCδ/PYK2 pathway in HPAEpiCs. To activate the activity of NADPH oxidase, it requires the stimulus-induced gp91*^phox^* membrane translocation and association with the Rac (small GTPase), p67*^phox^* and p47*^phox^*. In this process, activated p47*^phox^* translocates to the membrane and then recruited p67*^phox^* through the association between of the SH3 domain of p67*^phox^* and p47*^phox^* C-terminus. Thus, to be a switch role in ROS generation, p47*^phox^* mediates the activation of NADPH oxidase. In this study, according to the results of inhibition of p47*^phox^*, we proposed that S1P promotes p47*^phox^* translocation leading to activation of NADPH oxidase activity and ROS generation. The ROS play a role of mediators to regulate ICAM-1 expression on HPAEpiCs.

The role of NF-κB has been established in inflammatory responses. Extracellular stimuli are highly dependent on the various signal transductions to activate NF-κB, which regulates the transcription of several target genes. Several transcription factor response elements are identified on the ICAM-1 promoter and involved in the transcriptional regulation, including NF-κB (Roebuck and Finnegan, 1999; Yang C.M. et al., 2010; Hadad et al., 2011). These studies indicate that to activate NF-κB is a critical point in ICAM-1 expression within inflammatory responses. Our results indicated that S1P stimulates both nuclear translocation and phosphorylation of p65 in HPAEpiCs. Moreover, we found that blocking the signal transduction of NF-κB could attenuate S1P-induced ICAM-1 expression. Consistent with previous reports, our results indicated that NF-κB is regulated by various signaling components, such as ROS (Yuan et al., 2014), PKCδ (Lin et al., 2014), or PYK2 (Lee and Yang, 2013). These consistent results pointed out that in HPAEpiCs, S1P stimulated NF-κB p65 phosphorylation via a PKCδ/PYK2/NADPH oxidase/ROS pathway.

## Conclusion

In summary (**Figure 8**), our results indicated that challenge with S1P induces ICAM-1 expression on HPAEpiCs and facilitates monocyte adhesion. The ICAM-1 induction was mediated by Gq- and Go/i-coupled receptors/PKCδ/PYK2/NADPH oxidase/ROS-dependent NF-κB activation. These results provide new insights into the mechanisms of S1P-induced the expression of ICAM-1 and monocyte adhesion and thus exaggerate the inflammatory responses. Increased understanding of signaling mechanisms underlying ICAM-1 gene expression will promote the opportunities to develop the anti-inflammation therapeutic strategies.

## Author Contributions

CC, CC, RL, CY, LD, and CM substantially contributed to the conception or design of the work, the acquisition, analysis, and interpretation of data for the work. CC, CC, RL, CY, LD, and CM drafted the work and revised it critically for important intellectual content. CC, CC, RL, CY, LD, and CM finally approved the version to be published. CC, CC, RL, LD, CY, and CM agreed to be accountable for all aspects of the work in ensuring that questions related to the accuracy or integrity of any part of the work are appropriately investigated and resolved.

## Conflict of Interest Statement

The authors declare that the research was conducted in the absence of any commercial or financial relationships that could be construed as a potential conflict of interest.
